# The Effectiveness of mHealth Apps in the Rehabilitation of Children with Attention-deficit Hyperactivity Disorder

**Published:** 2018-01

**Authors:** Azadeh BASHIRI, Marjan GHAZISAEEDI

**Affiliations:** Dept. of Health Information Management, School of Allied Medical Sciences, Tehran University of Medical Sciences, Tehran, Iran

## Dear Editor-in-Chief

Regarding American Psychiatric Association, Attention-Deficit/Hyperactivity Disorder (ADHD) is one of the most common neurodevelopmental disorders in early childhood. The symptoms of ADHD include inattention, impulsivity, and hyperactivity. Such disorders lead to disruption in the educational, social and individual relationships areas and in other life aspects. Studies have highlighted the side effects of medications in the rehabilitation of individuals with ADHD and supported that many of children with ADHD despite pharmacological treatments experience cognitive dysfunctions (1,2). In recent years, growing interests in the use of mobile-Health applications in psychiatric and behavioral domains for delivering health care has combatted these issues. mHealth applications provide one of the best therapeutic strategies to improve the cognitive rehabilitation in the children with ADHD and enhance the quality of their life (3).

The goals of mHealth apps as one of the main subsets of e-health, are behavior change, education and diagnostic evaluations, collecting and reporting data, direct recording of health status, providing electronic decision supports, facilitating communication, planning and scheduling, alleviating the economic burden of diseases, enhancing the quality of health research and generally improving the effectiveness of healthcare services and health outcomes (4). mHealth apps on different platforms such as iOS and Android with low cost or free access, provide attractive and multilingual programs or games to help the children with ADHD in the information and time management, creativity promotion, making informed decisions and doing tasks, improving their relationships and habits such as insomnia. In addition, their capability improves problematic areas such as working memory, attention, concentration, prioritization, impulsivity, organizational skill, social relationship and educational progress in the children with ADHD (5, 6). Table 1 shows mHealth apps in improving the rehabilitation of children with ADHD and highlights their functionality, cost, and operating system. By investigating different mHealth apps about the children with ADHD, we categorized them into “Art Apps”, “Enhance Creativity Apps”, “Focus, Memory, Attention and Less Distraction Apps”, “Manage time Apps”, “Manage Information Apps”, “Sleep Apps”, and “Social Success Apps”. In 2016 the mHealth applications such as 30/30, Priority Matrix, Evernote, Dropbox, MindNode, MotivAider, EpicWin selected as the best apps in the rehabilitation of children with ADHD. The children with ADHD need to improve their relationships and behavior with the environment.

**Table 1:** Different mHealth apps in the rehabilitation of children with ADHD

***mHealthApps***	***Apps Name***	***Cost***	***OS***	***mHealthApps***	***Apps Name***	***Cost***	***OS***
*Art Apps*	Greatest Artists: Jigsaw Puzzle	Free to $4.99	Android, iOS	*Manage time Apps*	Todoist	Free to $29	iOS, Android, iOS
	How to Make Origam	Free	Android, iOS		Listastic	$2.99	
*Enhance Creativity Apps*	Hair Salon: Kids Games	Free	Android, iOS		Coach.me	Free	iOS, Android
	SimpleMind	Free to $5.99	iOS, Android		Finish 2Do	Free $44.99, $2.99	iOS iOS, Android
	Freedom	Free to $2.42	Android		TeuxDeux	Free	iOS
	Rescue Time	Free to $9	Android		EpicWin	$1.99	iOS
*Focus, memory, attention and Less Distraction Apps*	Focus@Will	Free to $8.33	iOS, Android		Evemote	Free	iOS, Android
	123 Tocken me	Free to $9.99	iOS		MIN TO GO	99 cent	iOS
	MotivAider	$1.99	iOS, Android		Priority Matrix	Free	iOS
	CogniFit Day	Free	iOS		White Noise	$1. 99	iOS, Android
	Elevate	Free	android, iOS	*Sleep Apps*	Relax Melodies	Free	iOS, Android
	Lumosity	Free	android, iOS		Deep Sleep with Andrew Johnson	$2. 99	iOS, Android
	Mind Node	9.99	iOS		Pzizz Sleep	Free	iOS
	Brain Training: Focus	Free	Android		To Bed	Free	iOS
	Eidetic for Long-Term Memory	Free	iOS		Sleep Bot	Free	iOS
	Fit Brains Trainer	Free	iOS		Sleep Cycle	Free	iOS
	iThoughts for Mind Mapping	11.99	iOS		Unstuck	Free	iOS, Android
	Brain Yoga for Relaxing Brain Training		Android, free		Sleep as Android	Free to $2.99	Android
	Evernote	Free to $24.99	iOS, Android		Chronos	Free	iOS, Android
*Manage Information Apps*	Mint	Free	iOS, Android		Podcast Players	Free to $3.99	iOS, Android
	Google Voice	Free	iOS, Android		How Would You Feel If	$1.99, $3.99	iOS, Android
	Boomerang for Gmail	free to $4.99	Android		Social Quest	$21.99	iOS
	Dropbox	Free to $8.25	iOS, Android	Social Success Apps	Model Me Going Places	Free	iOS
*Manage time Apps*	IFTTT (If This Then That)	Free	iOS, Android		Lets Be Social	$19.99	iOS
	30/30	Free	iOS		$39.99	iOS	
	Priority Matrix	Free to $8.25	iOS, Android		The Social Navigator	$4.99	iOS
	AutoSilent	$2.99	iOS, Android		Touch and Leam	$1.99	iOS
	FreakyAlarm	$1.99	iOS				
	Wake N Shake	$0.99	iOS				

The interactive nature of mHealth apps along with their capability to be adapted and customized based on individuals’ needs, leads in improving care, promoting rehabilitation and enhancing the quality of life.
